# Correlation Between Apolipoprotein B and Severity of Coronary Artery Stenosis in Individuals Aged 45 and Above: A Cross‐Sectional Study

**DOI:** 10.1155/cdr/2249959

**Published:** 2026-08-05

**Authors:** Dingchao Lv, Mao Ye, He Huang, Suqi Lv, Chen Li, Xiaolan Wang, Bin Yang

**Affiliations:** ^1^ The Second Hospital of Shanxi Medical University, Taiyuan, Shanxi Province, China, sxmu.edu.cn; ^2^ Department of Cardiology, The Second Hospital of Shanxi Medical University, Taiyuan, Shanxi Province, China, sxmu.edu.cn; ^3^ Department of Cardiovascular Medicine, Changzhi People′s Hospital, Changzhi, Shanxi Province, China; ^4^ The Third Clinical Medical College of Changzhi Medical College, Changzhi, Shanxi Province, China; ^5^ Changzhi People′s Hospital, Changzhi, Shanxi Province, China; ^6^ Department of General Medicine, Changzhi People′s Hospital, Changzhi, Shanxi Province, China

**Keywords:** Apolipoprotein B, coronary artery disease, Gensini score

## Abstract

**Background:**

Apolipoprotein B (ApoB) is widely recognized as a biomarker significantly associated with cardiovascular risk. However, the correlation between ApoB and the severity of coronary artery anatomical stenosis (Gensini score) in individuals aged 45 and above has not been fully studied.

**Methods:**

A cross‐sectional retrospective analysis was conducted, involving 818 patients who underwent coronary angiography. Fasting blood samples were collected to measure lipid parameters, including ApoB. The severity of coronary artery stenosis was assessed using the Gensini score, and patients were divided into two groups based on the median cut‐off (<36 vs. ≥ 36). A multivariate regression model was used to evaluate the association between ApoB and the severity of stenosis after adjusting for potential confounding factors.

**Results:**

Compared to the mild stenosis group (Gensini score < 36), the moderate‐to‐severe stenosis group (score ≥ 36) had significantly higher levels of ApoB (1.0 ± 0.3 vs. 0.9 ± 0.3 g/L) and LDL‐c (*p* < 0.05), while HDL‐c and ApoA levels were lower (*p* < 0.001). Multivariate regression analysis indicated that ApoB was independently associated with moderate‐to‐severe coronary stenosis (*β* = 46.1, 95% CI: 31.3–61.0 *p* < 0.001). Sensitivity analysis using continuous Gensini score confirmed the association (*β* = 22.57, 95% CI: 13.67–31.47, *p* < 0.0001). Dose–response analysis revealed that higher ApoB tertiles were associated with progressively higher Gensini scores (*P* for trend = 0.001). Stratified analysis revealed a consistent positive correlation between ApoB and Gensini scores across all clinical subgroups (all *p* < 0.05), with no significant interactions detected (all *P* for interaction > 0.05).

**Conclusion:**

ApoB levels are significantly positively correlated with the severity of coronary artery stenosis in the population aged ≥ 45 years, indicating its potential clinical application value in evaluation.

## 1. Introduction

Coronary artery disease (CAD) is a major global health issue, marked by high morbidity and mortality, primarily due to atherosclerosis. The Gensini score, which evaluates coronary artery stenosis severity, is crucial for predicting clinical outcomes like myocardial infarction and heart failure [1].

Apolipoprotein B (ApoB), a key structural element of atherogenic lipoproteins such as low‐density lipoprotein cholesterol (LDL‐c), VLDL‐c, and remnant particles, is considered a more accurate predictor of cardiovascular risk compared to traditional lipid measures. ApoB directly measures the number of atherogenic particles, offering a more precise assessment of their cumulative pathogenic potential compared with LDL‐c, which quantifies cholesterol content within individual lipoprotein classes [2].

Large‐scale epidemiological studies and meta‐analyses have demonstrated a strong association between elevated ApoB levels and cardiovascular events, independent of LDL‐c and other risk factors [3, 4]. Additionally, Mendelian randomization analyses support a causal relationship between ApoB and CAD, thereby underscoring its biological significance [5]. Although there is a wealth of literature documenting the predictive value of ApoB for cardiovascular outcomes [6], its precise relationship with the anatomical severity of coronary atherosclerosis, as measured by the Gensini score, has not yet been fully explored in specific populations such as those aged 45 years and over. Most existing studies have examined younger cohorts or mixed age groups, and fewer have specifically examined the correlation between ApoB and anatomical stenosis severity as quantified by the Gensini score. Furthermore, data on ApoB and coronary anatomy remain limited in Asian populations. This study addresses these gaps by examining the relationship between ApoB levels and coronary artery stenosis severity, as measured by the Gensini score, in individuals aged 45 and older—a specific population with distinct cardiovascular risk profiles.

## 2. Materials and Methods

### 2.1. Study Population

**Figure 1:**
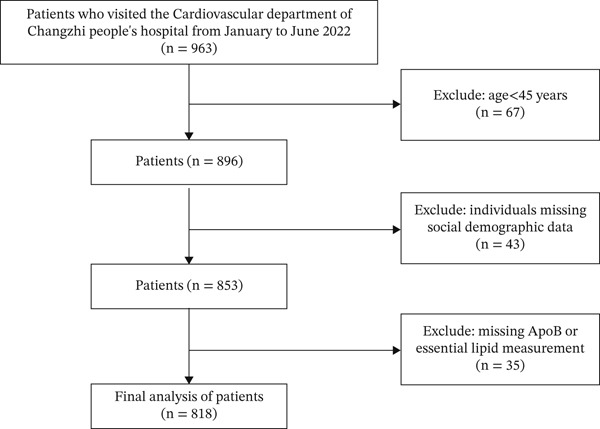
Flow diagram of study selection.

This cross‐sectional, retrospective study analyzes clinical data from 963 patients who underwent coronary angiography at Changzhi People′s Hospital′s Department of Cardiovascular Medicine between January and June 2022. Ultimately, 818 subjects with complete data were included. The study excluded participants based on the following criteria: (1) 67 patients younger than 45 years, (2) 43 individuals lacking social demographic data, and (3) 35 patients without ApoB or essential lipid measurements (Figure 1).

The study complied with the ethical guidelines outlined in the Declaration of Helsinki and its later revisions. It received approval from the Ethics Committee of Changzhi People′s Hospital (Ethics Review Approval Number: 2025K041).

### 2.2. Assessment of Exposure

Data were systematically collected on eligible patients, covering sociodemographic factors (age, sex, height, weight, body mass index, smoking status, and family medical history), health conditions (hypertension, diabetes mellitus, dyslipidemia, cardiovascular disease, and statin therapy), and laboratory tests for total cholesterol, high‐density lipoprotein cholesterol (HDL‐c), LDL‐c, Apolipoprotein A (ApoA), ApoB, creatinine, urea, and uric acid [7, 8]. Smokers were identified as individuals who had smoked for over 6 months or consumed more than 100 cigarettes in total, while drinkers were those who had at least 12 alcoholic beverages in the past year [9]. Hypertension was defined as a systolic blood pressure of ≥ 140 mmHg, diastolic blood pressure of ≥ 90 mmHg, self‐reported hypertension history, or antihypertensive medication use [10]. Diabetes mellitus was diagnosed based on a fasting blood glucose level ≥ 7.0 mmol/L, glycosylated hemoglobin ≥ 6.5*%*, a 2‐h postprandial or random glucose level ≥ 11.1 mmol/L during an oral glucose tolerance test, self‐reported diabetes history, or antihyperglycemic drug use [11]. Dyslipidemia was identified by meeting any of these criteria: TC ≥ 6.22 mmol/L, TG ≥ 2.26 mmol/L, LDL − c ≥ 4.14 mmol/L, HDL − c < 1.04 mmol/L, or a self‐reported history of the condition [12].

### 2.3. Blood Sampling and Laboratory Analysis

Fasting venous blood was collected at rest within 24 h of admission. Lipid profiles and fasting blood glucose were evaluated in the central laboratory on the sample collection day. Lipid profiles—including total cholesterol, triglycerides, HDL‐c, LDL‐c, ApoA, and ApoB—were measured using standard enzymatic and immunoturbidimetric methods. Renal function markers (urea, creatinine, and uric acid) and fasting blood glucose were also assessed using standard laboratory methods. A fully automated biochemical analyzer was used for all measurements.

### 2.4. Coronary Artery Stenosis Score Measurement

All participants underwent coronary angiography, and their Gensini scores were independently assessed by at least two interventional cardiologists to obtain an average score. Coronary stenosis severity was quantitatively evaluated following American Heart Association guidelines [13]. Coronary artery stenosis and lesion locations were evaluated using a scoring system: Stenosis ≤ 25*%* received a score of 1, 26%–50% was scored as 2, 51%–75% as 4, 76%–90% as 8, 91%–99% as 16, and total occlusion as 32 [14]. The significance of lesion locations within the coronary circulation varies, necessitating the application of specific location coefficients for accurate assessment. Lesions in the left coronary artery′s main trunk are weighted by a factor of 5. In contrast, lesions in the proximal, middle, and distal segments of the left anterior descending branch are weighted by factors of 2.5, 1.5, and 1, respectively. A coefficient of 2.5 is assigned for the proximal left circumflex artery, while a coefficient of 1 is assigned for the middle and distal segments of the circumflex artery, the first diagonal, obtuse marginal branch, right coronary artery, and posterior descending branch. The second diagonal branch and posterior lateral branch each have a coefficient of 0.5, along with other modifications [15]. The Gensini score quantifies coronary artery stenosis severity by summing products of coefficients based on stenosis severity and lesion location, with higher scores indicating greater severity [16, 17]. Patients were divided into two groups according to the median Gensini score (cutoff = 36), as previously established in cardiovascular risk stratification studies: 547 patients were placed in the mild stenosis group (Gensini score < 36), while 271 patients were categorized in the moderate‐to‐severe stenosis group (Gensini score ≥ 36) [18]. The median cutoff was selected based on established literature (Roy SS et al., Indian Heart J, 2018) and is widely used in cardiovascular risk stratification studies. Additionally [18], ApoB was categorized into tertiles (T1: < 0.77 g/L; T2: 0.77–1.00 g/L; and T3: > 1.00 g/L) to assess dose–response relationships. The term “Multivessel disease” refers to a narrowing of 70% or more in a minimum of two blood vessels, each measuring 2.25–5.75 mm in diameter, accompanied by a distinct implicating vessel [19].

### 2.5. Statistical Analysis

Participants were divided into two groups based on the median Gensini scores, and their baseline characteristics were compared. Continuous variables with a normal distribution were summarized as mean ± standard deviation, and *t*‐tests were used for comparing groups. Nonnormally distributed continuous variables were presented as median (25th percentile and 75th percentile), with intergroup comparisons conducted using the Mann–Whitney *U* test. Categorical variables were presented as frequencies (*n*) or percentages (%), and group comparisons were conducted using the chi‐square test. We utilized multivariable linear regression to assess the independent relationship between ApoB levels (per 1 g/L increase) and Gensini score (as a continuous variable) and multivariable logistic regression for the binary outcome (Gensini score < 36 vs. ≥ 36). Three models were constructed: unadjusted model: no covariates included; Model 1: adjusted for demographic factors (gender, age, and BMI); and model 2 (fully adjusted): adjusted for gender, age, BMI, total cholesterol, triglycerides, HDL‐c, LDL‐c, urea, uric acid, creatinine, ApoA, statin therapy, hypertension, diabetes, smoking, dyslipidemia, cardiovascular disease, and family history. Notably, ApoB was included only as the exposure variable, not as a covariate, to avoid overadjustment. Formal interaction testing was performed by including multiplicative interaction terms between ApoB and each stratification variable (statin therapy, diabetes, hypertension, CVD, hyperlipidemia, smoking, and family history). A two‐sided *p* value < 0.05 was considered statistically significant. Statistical analyses were performed using R software Version 4.3.3.

## 3. Results

### 3.1. Study Population and Baseline Characteristics

The baseline characteristics of 818 patients undergoing coronary angiography were evaluated. The cohort was divided into two groups according to the Gensini score: mild stenosis (score < 36, *n* = 547) and moderate‐to‐severe stenosis (score ≥ 36, *n* = 271). The analysis indicated that individuals with moderate‐to‐severe stenosis were significantly older (62.5 ± 8.8 years vs. 60.0 ± 8.8 years, *p* < 0.001), had a higher proportion of males (73.8% vs. 53.7%, *p* < 0.001), and showed a greater prevalence of diabetes mellitus (26.0% vs. 13.4%) and smoking history (49.8% vs. 33.9%) compared to those with mild stenosis (all *p* < 0.001). In lipid metabolism markers, individuals with moderate‐to‐severe stenosis showed significantly higher ApoB (1.0 ± 0.3 vs. 0.9 ± 0.3 g/L, *p* < 0.001) and LDL‐c levels (2.5 ± 0.9 vs. 2.4 ± 0.8 mmol/L, *p* = 0.009). Protective markers, HDL‐c (1.0 ± 0.3 vs. 1.1 ± 0.3 mmol/L) and ApoA (1.1 ± 0.2 vs. 1.3 ± 0.3 g/L), were significantly reduced in this group (both *p* < 0.001). The high stenosis group showed significantly elevated urea (5.5 ± 2.4 vs. 5.0 ± 1.8 mmol/L, *p* < 0.001) and creatinine levels (72.6 ± 28.7 vs. 68.1 ± 33.5 * μ*mol/L, *p* < 0.001) compared with the control group. Statin use was significantly less common in the high stenosis group (14.5%) compared with the other group (26.2%, *p* < 0.001). There were no statistically significant differences in BMI, uric acid levels, hypertension, cardiovascular disease, hyperlipidemia, or family history (all *p* > 0.05) (Table 1).

**Table 1:** Baseline characteristics according to the Gensini score.

Characteristic	Total (*n* = 818)	Gensini *s* *c* *o* *r* *e* < 36 (*n* = 547)	Gensini *s* *c* *o* *r* *e* ≥ 36 (*n* = 271)	*p* value
Age (years)	60.8 ± 8.9	60.0 ± 8.8	62.5 ± 8.8	< 0.001
BMI (kg/m^2^)	22.7 ± 8.5	22.6 ± 3.0	22.8 ± 4.3	0.187
Gender				
Male	494 (60.2%)	293 (53.7%)	200 (73.8%)	< 0.001
Female	326 (39.8%)	253 (46.3%)	71 (26.2%)	
Statin therapy				
Yes	182 (22.4%)	142 (26.2%)	39 (14.5%)	< 0.001
No	631 (77.6%)	401 (73.8%)	230 (85.5%)	
Diabetes				
Yes	143 (17.6%)	73 (13.4%)	70 (26.0%)	< 0.001
No	670 (82.4%)	470 (86.6%)	199 (74.0%)	
Hypertension				
Yes	450 (55.4%)	294 (54.1%)	155 (57.6%)	0.348
No	363 (44.6%)	249 (45.9%)	114 (42.4%)	
CVD				
Yes	145 (17.8%)	98 (18.0%)	47 (17.5%)	0.840
No	668 (82.2%)	445 (82.0%)	222 (82.5%)	
Hyperlipidemia				
Yes	168 (27.3%)	116 (28.7%)	52 (24.6%)	0.282
No	447 (72.7%)	288 (71.3%)	159 (75.4%)	
Smoking				
Yes	319 (39.2%)	184 (33.9%)	134 (49.8%)	< 0.001
No	494 (60.8%)	359 (66.1%)	135 (50.2%)	
Family history				
Yes	104 (12.8%)	69 (12.7%)	35 (13.0%)	0.903
No	709 (87.2%)	474 (87.3%)	234 (87.0%)	
TC (mmol/L)	4.0 ± 3.1	4.0 ± 3.3	4.0 ± 2.6	0.620
TG (mmol/L)	1.6 ± 1.0	1.6 ± 1.0	1.5 ± 0.9	0.654
HDL‐c (mmol/L)	1.1 ± 0.3	1.1 ± 0.3	1.0 ± 0.3	< 0.001
LDL‐c (mmol/L)	2.4 ± 0.8	2.4 ± 0.8	2.5 ± 0.9	0.009
Urea (mmol/L)	5.1 ± 2.0	5.0 ± 1.8	5.5 ± 2.4	< 0.001
Uric acid (*μ*mol/L)	295.1 ± 94.1	295.1 ± 94.1	309.0 ± 101.4	0.124
Creatinine (*μ*mol/L)	69.6 ± 32.0	68.1 ± 33.5	72.6 ± 28.7	< 0.001
ApoA (g/L)	1.3 ± 0.3	1.3 ± 0.3	1.1 ± 0.2	< 0.001
ApoB (g/L)	0.89 ± 0.27	0.9 ± 0.3	1.0 ± 0.3	< 0.001
Gensini score	30.4 ± 38.8	8.0 ± 10.0	75.2 ± 36.1	< 0.001

*Note:* Normally distributed continuous variables are expressed as mean ± SD. Categorical variables are expressed as *n* (%). *p* value < 0.05.

Abbreviations: ApoA, Apolipoprotein A; ApoB, Apolipoprotein B; BMI, body mass index; CVD, cardiovascular disease; HDL‐c, high‐density lipoprotein cholesterol; LDL‐c, low‐density lipoprotein cholesterol; TC, total cholesterol; TG, triglyceride.

### 3.2. The Association Between ApoB and the Degree of Coronary Stenosis

A multifactorial regression analysis revealed that ApoB levels are independently associated with the severity of coronary artery stenosis, as indicated by the Gensini score, while being influenced by several confounding factors.

#### 3.2.1. Continuous Gensini Score Analysis

In the unadjusted model, each 1 g/L increase in ApoB was associated with a significant 28.5‐point rise in the Gensini score (95% CI: 20.0–37.1, *p* < 0.001). After controlling for sex, age, and body mass index in Model 1, the Gensini score rose to 32.8 points (95% CI: 24.6–41.0, *p* < 0.001). After fully adjusting for lipid profiles, renal function markers, comorbidities, treatment history, lifestyle factors, and family history (Model 2), the Gensini score increased to 46.1 points (95% CI: 31.3–61.0, *p* < 0.001) (Table 2).

**Table 2:** Multivariate regression analysis of Apolipoprotein B and the degree of coronary stenosis (Gensini score).

Variable	*β*(95% CI), *p* value		
	Nonadjusted	Model 1	Model 2
Continuous Gensini score			
ApoB (per 1 g/L)	28.5 (20.0, 37.1), < 0.001	32.8 (24.6, 41.0), < 0.001	46.1 (31.3, 61.0), < 0.001
	**OR (95% CI), p value**		
Binary Gensini (≥ 36 vs. < 36)			
ApoB (per 1 g/L)	2.23 (1.29, 3.84), 0.004	4.04 (2.19, 7.45), < 0.0001	1.15 (1.03, 1.28), 0.015

*Note:* ApoB was included only as the exposure variable, not as an adjusting covariate, in Model 2. Nonadjusted model: No covariates. Model 1: Adjusted for gender, age, and BMI. Model 2 (fully adjusted): Adjusted for gender, age, BMI, TC, TG, HDL‐c, LDL‐c, urea, uric acid, creatinine, ApoA, statin therapy, hypertension, diabetes, smoking, dyslipidemia, CVD, and family history. *p* value < 0.05.

Abbreviations: *β*, regression coefficient; CI, confidence interval; OR, odds ratio.

#### 3.2.2. Binary Gensini Score Analysis (≥ 36 vs. < 36)

In the unadjusted model, each 1 g/L increase in ApoB was associated with an odds ratio of 2.23 (95% CI: 1.29–3.84, *p* = 0.004) for moderate‐to‐severe stenosis. After adjusting for demographic factors in Model 1, the OR increased to 4.04 (95% CI: 2.19–7.45, *p* < 0.0001). In Model 2 (fully adjusted), the OR was 1.15 (95% CI: 1.03–1.28, *p* = 0.015), indicating a statistically significant positive correlation (Table 2).

#### 3.2.3. Dose–Response Analysis

Stratified analysis by ApoB tertiles revealed a significant dose–response relationship between ApoB levels and Gensini score. Compared with the lowest tertile (T1, ApoB < 0.77 g/L), both T2 (ApoB 0.77–1.00 g/L) and T3 (ApoB > 1.00 g/L) showed significantly higher Gensini scores (T2: 33.97, 95% CI: 29.44–38.50, *p* < 0.01 and T3: 33.95, 95% CI: 29.29–38.61, *p* < 0.01 vs. T1: 23.49, 95% CI: 18.97–28.00). The *P* for trend was 0.001, confirming a robust linear association between ApoB levels and coronary stenosis severity (Table S1).

### 3.3. Stratified Analysis of ApoB and Gensini Score

Stratified regression analysis revealed a consistent positive correlation between ApoB levels and Gensini scores across different clinical subgroups, with the strength of this association affected by certain factors. Formal interaction testing was performed for all stratification variables. No significant interactions were observed between ApoB and any clinical subgroup, including statin therapy (*P* = 0.393), diabetes (*P* = 0.796), hypertension (*P* = 0.127), CVD (*P* = 0.872), hyperlipidemia (*P* = 0.704), smoking (*P* = 0.471), or family history (*P* = 0.237). The overall pooled effect was beta = 22.57 (95% CI: 13.67–31.47), *p* < 0.0001, suggesting a consistent positive association across all clinical subgroups (Table 3). Within the gender stratification, the association between ApoB and Gensini scores was more pronounced in male patients (*β* = 34.6, 95% CI: 22.9–46.4) compared with female patients (*β* = 25.6, 95% CI: 14.0–37.2). In the metabolism‐related subgroup analysis, individuals without comorbid hyperlipidemia (*β* = 43.5, 95% CI: 30.7–56.3) and those not undergoing statin therapy (*β* = 27.1, 95% CI: 17.0–37.2) demonstrated stronger ApoB–stenosis associations. Furthermore, the association was more robust in patients with a history of comorbid hypertension (*β* = 31.6, 95% CI: 20.8–42.5) or without cardiovascular disease (*β* = 32.0, 95% CI: 22.0–41.9), whereas it was relatively diminished in smokers (*β* = 24.6, 95% CI: 10.6–38.5) and individuals with diabetes (*β* = 25.3, 95% CI: 2.6–48.1). Individuals with a positive family history exhibited the most pronounced ApoB effect value (*β* = 34.4, 95% CI: 14.3–54.5) (Table 3).

**Table 3:** Stratified analysis of ApoB and the Gensini score.

Stratification variable	Total	*β*(95% CI)	*p* value	*P* for interaction
Overall	818	22.57 (13.67, 31.47)	< 0.0001	—
Gender				
Male	493	34.6 (22.9, 46.4)	< 0.0001	0.393
Female	324	25.6 (14.0, 37.2)	< 0.0001	
Statin therapy				
Yes	181	11.6 (−10.4, 33.7)	0.302	0.393
No	631	27.1(17.0, 37.2)	< 0.0001	
Diabetes				
Yes	143	25.3 (2.6, 48.1)	0.0309	0.796
No	669	29.3 (20.0, 38.4)	< 0.0001	
Hypertension				
Yes	449	31.6 (20.8, 42.5)	< 0.0001	0.127
No	363	25.4 (11.5, 39.4)	0.0004	
CVD				
Yes	145	19.3 (2.9, 35.7)	0.0224	0.872
No	667	32.0 (22.0, 41.9)	< 0.0001	
Hyperlipidemia				
Yes	168	21.5 (10.6, 38.5)	0.0303	0.704
No	447	43.5 (30.7, 56.3)	< 0.0001	
Smoking				
Yes	318	24.6 (10.6, 38.5)	0.0006	0.471
No	494	32.7 (21.9, 43.4)	< 0.0001	
Family history				
Yes	104	34.4 (14.3, 54.5)	0.0011	0.237
No	708	28.8 (19.4, 38.2)	< 0.0001	

*Note:* All models adjusted for age, BMI, and gender. *p* value < 0.05.

Abbreviation: CI, confidence interval.

## 4. Discussion

This study demonstrated that higher ApoB levels are independently associated with increased coronary artery stenosis severity, as measured by the Gensini score, in individuals aged 45 and older. Multivariate analyses revealed a dose–response relationship, with the most pronounced effects observed after adjusting for lipid profiles, comorbidities, and therapeutic interventions (*β* = 46.1, 95% CI: 31.3–61.0, *p* < 0.001 for continuous Gensini; OR = 4.04, *p* < 0.0001 for binary Gensini ≥ 36 in Model 1). Further stratification indicated no significant effect modification by clinical factors, with a consistent positive association observed across all subgroups (all *P* for interaction > 0.05).

Our results support recent evidence indicating that ApoB is a superior predictor of atherosclerotic burden compared with traditional lipid parameters [20, 21]. The PROVE‐IT TIMI 22 trial demonstrated that ApoB is a stronger predictor of residual cardiovascular risk than LDL‐c in patients on statin therapy [22], supporting our study′s findings of potentially attenuated ApoB association in statin users. Notably, the stratified analysis showed that the ApoB–stenosis association was attenuated in statin users (beta = 11.64, *p* = 0.302) compared with nonusers (beta = 22.14, *p* < 0.0001), suggesting that lipid‐lowering therapy may modify the pathogenic effects of ApoB. However, the lower prevalence of statin use in the severe stenosis group (14.5% vs. 26.2%) raises concerns about indication bias. This disparity may reflect differences in baseline cardiovascular risk profiles, prior statin exposure before hospitalization, or other confounding factors that could influence the observed association. Residual confounding from unmeasured factors cannot be entirely excluded. The INTERHEART study reported a weaker correlation of ApoB in the Asian population, which may be attributed to ethnic variations in lipid metabolism or differences in statin use [23]. Our study provides valuable data from a Chinese population, contributing to the understanding of ethnic variations in ApoB‐related cardiovascular risk. Sex differences, with a higher *β* observed in men, were consistent with findings from the Frachial cohort, where ApoB‐driven risk was more pronounced in males, potentially due to androgen‐mediated lipid oxidation pathways [24]. Significantly, our novel association between ApoB and renal biomarkers extends previous research on accelerated atherosclerosis in chronic kidney disease (CKD), suggesting that ApoB may act as a mediator in the “cardiorenal metabolic syndrome” paradigm [25].

The robust correlation between ApoB and stenosis may originate from ApoB′s direct involvement in atherogenesis. ApoB, the main structural protein in LDL‐c and VLDL‐c particles, promotes endothelial retention of cholesterol‐rich lipoproteins, triggering foam cell formation and plaque progression [20, 26, 27]. The amplification of this effect after adjusting for LDL indicates that the number of atherogenic particles linked to ApoB exceeds their cholesterol content, confirming that ApoB particle quantity primarily determines cholesterol deposition in the arterial wall. Renal insufficiency, characterized by elevated levels of urea and creatinine, may exacerbate vascular injury through toxin‐induced oxidative stress and chronic inflammation. This process can generate ApoB‐driven feed‐forward loops that promote lipid deposition. Oxidatively modified ApoB particles, such as oxidized LDL‐c, can activate immune responses and lead to endothelial dysfunction, thereby further contributing to arterial inflammation and lipid peroxidation [28, 29]. The observed familial susceptibility, indicated by the *β* coefficient in the positive family history subgroup, suggests that genetic polymorphisms, such as APOBrs693, may influence the atherogenic potential of ApoB [27]. Clinical interpretation of effect size: To provide clinical context for the observed association, a 1 g/L increase in ApoB corresponds to approximately a 46‐point increase in Gensini score in Model 2, representing a shift from mild to moderate‐to‐severe stenosis categories. In the continuous analysis, the pooled beta coefficient of 22.57 (95% CI: 13.67–31.47) provides a more conservative estimate. The tertile analysis demonstrates that moving from T1 (ApoB < 0.77 g/L, Gensini = 23.5) to higher tertiles (T2 and T3: Gensini approximately 34.0) represents a clinically meaningful difference of approximately 10–11 points on the Gensini scale.

This study possesses several limitations. First, the cross‐sectional design restricts causal inference, highlighting the need for prospective cohort studies to confirm ApoB′s predictive value. Second, despite extensive efforts, residual confounding from unmeasured factors like dietary habits and physical activity may still exist. Third, the clinical indication for coronary angiography (ACS vs. CCS) was not systematically recorded, introducing potential confounding, as plaque characteristics differ substantially between these presentations. Fourth, the study sample mainly consists of individuals from hospitals in certain regions, which may introduce geographic bias and limit the generalizability of the findings.

Future research should concentrate on (1) interventional trials evaluating the effects of ApoB‐targeted treatments, like PCSK9 inhibitors, on stenosis progression, (2) mechanistic studies investigating the ApoB and renal insufficiency interaction, and (3) multicenter cohort studies incorporating multiorganomic data to identify susceptibility modifiers.

## 5. Conclusions

The study identifies a notable positive correlation between ApoB levels and coronary artery stenosis severity (Gensini score) in individuals aged 45 and older, highlighting the clinical significance of ApoB as a potential risk stratification biomarker in this population.

## Author Contributions

Mao Ye, Xiaolan Wang, and Dingchao Lv designed the study and conceived the paper. Bin Yang, Mao Ye, and Dingchao Lv performed statistical analysis and drafted the manuscript. Suqi Lv, He Huang, and Chen Li arranged the data and assisted with figure preparation. Dingchao Lv, Xiaolan Wang, and Bin Yang critically revised the manuscript.

## Funding

No funding was received for this manuscript.

## Disclosure

All authors read and approved the final manuscript.

## Ethics Statement

The following information was supplied relating to ethical approvals (i.e., approving body and any reference numbers): approved by the Ethics Committee of Changzhi People′s Hospital (Ethics Review Approval Number: 2025K041).

## Conflicts of Interest

The authors declare no conflicts of interest.

## Supporting information


**Supporting Information** Additional supporting information can be found online in the Supporting Information section. Table S1: Dose–response analysis of Apolipoprotein B tertiles and the Gensini score. Apolipoprotein B (ApoB) was divided into three tertiles: T1 (< 0.77 g/L), T2 (0.77–1.00 g/L), and T3 (> 1.00 g/L). This table lists the Gensini scores for each tertile, along with their 95% confidence intervals and the *p* values for the trends.

## Data Availability

The datasets used in this study are available from the corresponding author upon reasonable request.
