# Supplementary material for: Correlation Between Apolipoprotein B and Severity of Coronary Artery Stenosis in Individuals Aged 45 and Above: A Cross‐Sectional Study
Source: Cardiovasc Ther. 2026 Aug 5;2026:2249959. doi: 10.1155/cdr/2249959 (PMC13443114; doi:10.1155/cdr/2249959)
Supplement: Supplementary file 1 [file CDR-2026-2249959-s001.docx]

**Supplementary Table 1** Dose-response analysis: ApoB tertiles and Gensini score

| **ApoB Tertile** | **n (%)** | **β (95% CI)** | ***P-value vs. T1*** |
| --- | --- | --- | --- |
| T1 (Low, <0.77 g/L) | 280 (34.10%) | 23.49 (18.97, 28.00) | Reference |
| T2 (Middle, 0.77–1.00 g/L) | 278 (33.86%) | 33.97 (29.44, 38.50) | <0.01 |
| T3 (High, >1.00 g/L) | 263 (32.03%) | 33.95 (29.29, 38.61) | <0.01 |
| P for trend | 0.001 | | |

CI: Confidence interval.
